# Supplementary material for: Countering misinformation via WhatsApp: Preliminary evidence from the COVID-19 pandemic in Zimbabwe
Source: PLoS One. 2020 Oct 14;15(10):e0240005. doi: 10.1371/journal.pone.0240005 (PMC7556529; doi:10.1371/journal.pone.0240005)
Supplement: S1 Appendix — (PDF) [file pone.0240005.s001.pdf]

S1 Appendix. Messages.

Week 1:

With only 9 confirmed cases in Zimbabwe, and given the hardship lockdown imposes on people, many are questioning whether a 21 day lockdown is necessary, and what government's plan is in the longer term. But, it is possible to have the Coronavirus and not show any symptoms. At least 25% of people who have Coronavirus never show symptoms. This means you could catch it from someone who does not know they are sick, and you could unknowingly pass it on to other people, without even realising you were carrying it. This graphic visually demonstrates how physical distancing can help to contain the spread Covid-19.

Week 2:

Social media features a lot of false information about Coronavirus. One myth encourages people to breathe steam or drink hot water to kill Coronavirus. Importantly, **neither breathing hot steam nor drinking hot water kills the virus**. There is no miracle cure and researchers are doing their best to find something quickly, but it will take time. The best recommendations to avoid getting sick and to stop you spreading the virus are to:

- practise **physical distancing**
- **hand wash** thoroughly and frequently (with soap on your hands for 20 seconds)
- **wash surfaces regularly and well**, ideally with bleach or other disinfectant

You can read more here: `https://bit.ly/34rG14b`
