# Supplementary material for: Countering misinformation via WhatsApp: Preliminary evidence from the COVID-19 pandemic in Zimbabwe
Source: PLoS One. 2020 Oct 14;15(10):e0240005. doi: 10.1371/journal.pone.0240005 (PMC7556529; doi:10.1371/journal.pone.0240005)
Supplement: S2 Appendix — (PDF) [file pone.0240005.s002.pdf]

S2 Appendix. Coding decisions.

Main treatment variables:

Treatment: Coded as "1" if the WhatsApp broadcast list is assigned to the treatment condition. Treatment assignment varies each week.

Long experimental list: Coded as "1" if the WhatsApp broadcast list is assigned to the long experimental list. Experimental list assignment varies each week.

Main outcome variables:

Knowledge: In **week 1**, there are two questions that test respondent knowledge of the treatment messaging (see Week 1, Q4 and Q5 in S3 Appendix for exact wording). We code whether the respondent selected the correct responses, and *Knowledge* is coded as the standardized index, or z-score, of these two variables. In **week 2**, there is one question that tests respondent knowledge. The question allows for multiple options, meaning that there are four potentially correct responses (see Week 2, Q4 in S3 Appendix for exact wording). We code whether the respondent answered correctly for each option, and *Knowledge* is coded as the z-score of all four options.

Behavior: Coded based on how many activities on the experimental list that respondents received they mention that they participated in the last three days. The short experimental list had four options and responses are coded from "0" to "4", while the long list has five options and responses are therefore coded from "0" to "5".

Other variables:

Qualtrics: Coded as "1" if the individual responded through the Qualtrics link and "0" if the individual responded directly through WhatsApp.

Urban: Coded as "1" if the individual responded to living in the following districts: Harare, Bulawayo, Chitungwiza, Mutare, Gweru, Chinhoye, Masvingo, Kwekwe, Kadoma, and Norton.

Female: Coded as "1" if the individual indicated that they were female.

Months subscribed: The number of months that the WhatsApp broadcast list has been active, counting backward from April 2020.

WhatsApp broadcast list response rate %: The number of responses per week from a WhatsApp broadcast list, divided by the total number of individuals in that list.
