# Supplementary material for: Countering misinformation via WhatsApp: Preliminary evidence from the COVID-19 pandemic in Zimbabwe
Source: PLoS One. 2020 Oct 14;15(10):e0240005. doi: 10.1371/journal.pone.0240005 (PMC7556529; doi:10.1371/journal.pone.0240005)
Supplement: S3 Appendix — (PDF) [file pone.0240005.s003.pdf]

S3 Appendix. Survey questions used.

Week 1

Hello! Researchers from Harvard University are helping Kubatana to assess the impact of the messages we share. Please could you answer the **5 short questions** in their survey? The survey will take you **less than three minutes** to complete, and your answers will be anonymous. To participate, you need to be over 18. You can read the questions below and reply us directly on WhatsApp, OR you can fill in their survey online here:

1. Where are you located? [Indicate your city or district.]
2. What is your gender?
   (a) Female
   (b) Male
3. In the last 3 days, **HOW MANY** of the following activities did you perform? [Indicate the **TOTAL NUMBER** of activities, not the actual activities]

- Watched TV or listened to the radio
- Spoke to friends or family on the phone or WhatsApp
- **Visited a friend or family member**
- Went grocery shopping
- Received or earned money

Answer: [Indicate the **TOTAL NUMBER** of activities from 0 to 5]

4. To the best of your knowledge, approximately, how many people infected with CORONAVIRUS never show symptoms? [Choose a single response.]
   (a) 0%
   (b) **25%**
   (c) 50%
   (d) 75%
   (e) Do not know
5. To the best of your knowledge, if people implement physical distancing by cutting their exposure to others in half, how will this change the spread of the virus? [Choose a single response.]
   (a) Physical distancing makes no difference.
   (b) Half as many people will be infected.
   (c) A quarter as many people will be infected.
   (d) **Physical distancing will almost eliminate the spread of the virus.**
   (e) Do not know

Week 2

Hello! Thank you everyone for responding to our survey last week. This week again, researchers from Harvard University are helping Kubatana to assess the impact of the messages we share. Please could you answer the **5 short questions** in their survey? The survey will take you **less than three minutes** to complete, and your answers will be **anonymous**. To participate, you need to be over 18. You can read the questions below and reply us **by noon on Sunday** directly on WhatsApp, OR you can fill in their survey online here:

1. Where are you located? [Indicate your city or district.]
2. What is your gender?
   (a) Female
   (b) Male
3. In the last 3 days, **HOW MANY** of the following activities did you perform? [Indicate the **TOTAL NUMBER** of activities, not the actual activities]
   - Watched TV or listened to the radio
   - Spoke to friends or family on the phone or WhatsApp
   - **Visited a friend or family member**
   - Went grocery shopping

- Received or earned money

Answer: [Indicate the **TOTAL NUMBER** of activities from 0 to 5]

4. To the best of your knowledge, which of the following strategies most effectively deal with CORONAVIRUS? [Choose **ALL RESPONSES** that you think apply.]

   - Drinking hot water
   - Eating garlic, ginger, lemon and herbs
     Note: This information was not part of the messaging, and is thus not included in the coding for Knowledge.
   - **Hand washing with soap**
   - Inhaling hot steam
   - **Washing surfaces with bleach or other disinfectant**
   - None of these

Week 3

In the past three days, which of the following sources have you consulted about CORONAVIRUS? [Choose **ALL RESPONSES** that you think apply]

- Messages from family and friends
- Messages from the Government
- Messages from international organizations and agencies
- Messages from local civil society organisations or NGOs
- Messages that mention a news source
- Messages that mention a doctor as a source
- Messages that mention a government source
- None of these
