# Supplementary material for: Countering misinformation via WhatsApp: Preliminary evidence from the COVID-19 pandemic in Zimbabwe
Source: PLoS One. 2020 Oct 14;15(10):e0240005. doi: 10.1371/journal.pone.0240005 (PMC7556529; doi:10.1371/journal.pone.0240005)
Supplement: S4 Appendix — (PDF) [file pone.0240005.s004.pdf]

S4 Appendix. Estimation.

We estimate effects on knowledge using Equation (1):

$$y_{ilw} = \beta T_{lw} + \mu_w + \eta_b + \epsilon_{ilw}, \quad (1)$$

where outcome y for respondent i in broadcast list l in week w is regressed onto the treatment indicator T for WhatsApp broadcast list l in week w and week fixed effects. We additionally include either randomization block fixed effects η_b or, more demandingly, WhatsApp broadcast list fixed effects η_l. We cluster standard errors at the WhatsApp broadcast list-week level, since this is the level of randomized treatment assignment. As a result, there are more clusters than broadcast lists. β in Equation (1) estimates the causal effect of a WhatsApp message on knowledge.

We estimate effects on behavior using Equation (2):

$$y_{ilw} = \beta_1 T_{lw} + \beta_2 L_{lw} + \beta_3 (T_{lw} \times L_{lw}) + \mu_w + \eta_b + \epsilon_{ilw}, \quad (2)$$

where outcome y for respondent i in WhatsApp broadcast list l in week w is regressed onto the treatment indicator T for broadcast list l in week w, the list experiment indicator L for broadcast list l in week w, and the interaction of the two. We additionally include either randomization block fixed effects η_b or, more demandingly, WhatsApp broadcast list fixed effects η_l. Standard errors are clustered at the broadcast list-week level. β_1 in Equation (2) estimates treatment effects on the number of activities reported among respondents receiving the short experimental list; β_2 estimates the effect of receiving the long experimental list on the number of activities reported among those assigned to control, and β_3 estimates how the number of activities reported among respondents receiving the long experimental list varies between those assigned to the treatment as opposed to the control condition. β_3, therefore, estimates the causal effect of a WhatsApp message on behavior.
