# Supplementary material for: Countering misinformation via WhatsApp: Preliminary evidence from the COVID-19 pandemic in Zimbabwe
Source: PLoS One. 2020 Oct 14;15(10):e0240005. doi: 10.1371/journal.pone.0240005 (PMC7556529; doi:10.1371/journal.pone.0240005)
Supplement: S1 Table — (PDF) [file pone.0240005.s005.pdf]

S1 **Table.** Summary Statistics

	Obs.	Mean	SD	Min	Max
**Main treatment variables:**					
Treatment	868	0.52	0.50	0.00	1.00
Long list in list experiment	868	0.49	0.50	0.00	1.00
**Main outcome variables:**					
Knowledge	864	0.01	1.00	-2.03	1.85
Behavior	861	2.64	0.90	0.00	5.00
**Correct response to knowledge questions:**					
Week 1:					
25% of infected are symptomless	583	0.36	0.48	0.00	1.00
Distancing cuts infection rates almost entirely	570	0.83	0.38	0.00	1.00
Week 2:					
Drinking hot water helps	283	0.30	0.46	0.00	1.00
Hand washing with soap helps	283	0.70	0.46	0.00	1.00
Inhaling hot steam helps	283	0.25	0.43	0.00	1.00
Washing surface with disinfectant helps	283	0.56	0.50	0.00	1.00
**Other variables:**					
Qualtrics	868	0.18	0.39	0.00	1.00
Urban	868	0.76	0.43	0.00	1.00
Female	868	0.45	0.50	0.00	1.00
Months subscribed	868	20.63	19.41	1.00	76.00
WhatsApp broadcast list response rate (%)	868	0.02	0.02	0.00	0.17
