# Supplementary material for: Countering misinformation via WhatsApp: Preliminary evidence from the COVID-19 pandemic in Zimbabwe
Source: PLoS One. 2020 Oct 14;15(10):e0240005. doi: 10.1371/journal.pone.0240005 (PMC7556529; doi:10.1371/journal.pone.0240005)
Supplement: S2 Table — (PDF) [file pone.0240005.s006.pdf]

S2 **Table.** Balance

	Qualtrics	Urban	Female	List Time	Response Rate
**Panel A:**					
Treatment	0.01	0.03	0.00	-0.25	-0.00
	(0.03)	(0.02)	(0.03)	(0.15)	(0.00)
**Panel B:**					
Treatment	-0.00	0.04	0.02	0.00	-0.00
	(0.03)	(0.03)	(0.04)	(.)	(0.00)
Clusters	197	197	197	197	197
Observations	868	868	868	868	868

All specifications include week of intervention fixed effects. Panel A also includes randomization block fixed effects, while Panel B includes WhatsApp broadcast list fixed effects instead. Standard errors are clustered at week-list level. * $p < 0.1$, ** $p < 0.05$, *** $p < 0.01$.
