# Supplementary material for: Countering misinformation via WhatsApp: Preliminary evidence from the COVID-19 pandemic in Zimbabwe
Source: PLoS One. 2020 Oct 14;15(10):e0240005. doi: 10.1371/journal.pone.0240005 (PMC7556529; doi:10.1371/journal.pone.0240005)
Supplement: S3 Table — (PDF) [file pone.0240005.s007.pdf]

S3 **Table.** Knowledge

	All		Female		Male		Urban		Rural	
	No controls	Controls	No controls	Controls	No controls	Controls	No controls	Controls	No controls	Controls
**Panel A:**										
Treatment	0.26***	0.27***	0.36***	0.36***	0.19**	0.21**	0.26***	0.26***	0.36**	0.39***
	(0.06)	(0.06)	(0.11)	(0.11)	(0.08)	(0.08)	(0.07)	(0.07)	(0.15)	(0.15)
**Panel B:**										
Treatment	0.45***	0.45***	0.49***	0.49***	0.55***	0.54***	0.45***	0.46***	0.62**	0.62**
	(0.09)	(0.09)	(0.16)	(0.16)	(0.14)	(0.14)	(0.12)	(0.12)	(0.29)	(0.29)
Clusters	197	197	140	140	164	164	172	172	115	115
Observations	864	864	393	393	471	471	656	656	208	208

All specifications include week of intervention fixed effects. Panel A also includes randomization block fixed effects, while Panel B includes WhatsApp broadcast list fixed effects instead. Controls are indicators for Qualtrics response, urban, and female respondents. Standard errors are clustered at week-list level. * $p < 0.1$, ** $p < 0.05$, *** $p < 0.01$.
