# Supplementary material for: Countering misinformation via WhatsApp: Preliminary evidence from the COVID-19 pandemic in Zimbabwe
Source: PLoS One. 2020 Oct 14;15(10):e0240005. doi: 10.1371/journal.pone.0240005 (PMC7556529; doi:10.1371/journal.pone.0240005)
Supplement: S4 Table — (PDF) [file pone.0240005.s008.pdf]

S4 **Table.** Behavior

	All		Female		Male		Urban		Rural	
	No controls	Controls	No controls	Controls	No controls	Controls	No controls	Controls	No controls	Controls
**Panel A:**										
Treatment	0.32***	0.32***	0.35***	0.35***	0.30**	0.29**	0.33***	0.34***	0.26	0.26
	(0.08)	(0.07)	(0.10)	(0.10)	(0.12)	(0.12)	(0.09)	(0.09)	(0.18)	(0.18)
Long	0.37***	0.37***	0.33***	0.34***	0.38***	0.38***	0.34***	0.35***	0.32**	0.34**
	(0.08)	(0.08)	(0.11)	(0.11)	(0.13)	(0.13)	(0.10)	(0.10)	(0.15)	(0.15)
Treatment $\times$ Long	-0.30**	-0.31**	-0.33*	-0.34*	-0.28	-0.27	-0.27*	-0.28*	-0.26	-0.27
	(0.13)	(0.12)	(0.18)	(0.18)	(0.19)	(0.18)	(0.15)	(0.15)	(0.25)	(0.25)
α(Long + T $\times$ Long $\neq$ 0)	0.43	0.45	1.00	0.99	0.38	0.35	0.50	0.54	0.72	0.70
**Panel B:**										
Treatment	0.29***	0.28***	0.30*	0.30*	0.21*	0.19	0.30**	0.30**	0.22	0.20
	(0.09)	(0.09)	(0.17)	(0.17)	(0.12)	(0.13)	(0.12)	(0.12)	(0.26)	(0.27)
Long	0.46***	0.45***	0.52***	0.52***	0.48***	0.46***	0.47***	0.46***	0.31	0.29
	(0.09)	(0.09)	(0.12)	(0.12)	(0.15)	(0.15)	(0.12)	(0.12)	(0.23)	(0.24)
Treatment $\times$ Long	-0.40***	-0.39***	-0.39**	-0.39**	-0.43**	-0.41**	-0.35**	-0.34**	-0.35	-0.31
	(0.12)	(0.12)	(0.19)	(0.19)	(0.19)	(0.20)	(0.15)	(0.15)	(0.29)	(0.29)
α(Long + T$\times$Long $\neq$ 0)	0.47	0.42	0.34	0.34	0.69	0.68	0.19	0.19	0.86	0.91
Clusters	197	197	140	140	165	165	172	172	115	115
Observations	861	861	390	390	471	471	655	655	206	206

All specifications include week of intervention fixed effects. Panel A also includes randomization block fixed effects, while Panel B includes WhatsApp broadcast list fixed effects instead. Controls are indicators for Qualtrics response, urban, and female respondents. α(Long + Treatment$\times$Long $\neq$ 0) provides the p-value of the joint hypothesis that Long + Treatment$\times$Long $\neq$ 0. Standard errors are clustered at week-list level. * $p < 0.1$, ** $p < 0.05$, *** $p < 0.01$.
