# Supplementary material for: Countering misinformation via WhatsApp: Preliminary evidence from the COVID-19 pandemic in Zimbabwe
Source: PLoS One. 2020 Oct 14;15(10):e0240005. doi: 10.1371/journal.pone.0240005 (PMC7556529; doi:10.1371/journal.pone.0240005)
Supplement: S5 Table — (PDF) [file pone.0240005.s009.pdf]

S5 **Table.** Outcomes by week

	Week 1		Week 2	
	No controls	Controls	No controls	Controls
**Panel A:**				
Treatment	0.21***	0.21***	0.33**	0.37***
	(0.06)	(0.07)	(0.13)	(0.12)
**Panel B:**				
Treatment	0.37***	0.38***	0.36**	0.34**
	(0.10)	(0.10)	(0.15)	(0.14)
Long	0.41***	0.41***	0.41**	0.40**
	(0.10)	(0.09)	(0.18)	(0.18)
Treatment × Long	-0.45***	-0.46***	-0.32	-0.30
	(0.15)	(0.14)	(0.29)	(0.29)
α(Long + T×Long $\neq$ 0)	0.65	0.58	0.65	0.56
Clusters	110	110	87	87
Observations	581	581	280	280

All specifications include week of intervention fixed effects. Panel A also include randomization block fixed effects, while Panel B includes WhatsApp broadcast list fixed effects instead. Controls are indicators for qualtrics, urban, and female respondents. α(Long + Treatment×Long $\neq$ 0) provides the p-value of the joint hypothesis that Long + Treatment×Long $\neq$ 0. Standard errors are clustered at week-list level. * $p < 0.1$, ** $p < 0.05$, *** $p < 0.01$.
