# Supplementary material for: Countering misinformation via WhatsApp: Preliminary evidence from the COVID-19 pandemic in Zimbabwe
Source: PLoS One. 2020 Oct 14;15(10):e0240005. doi: 10.1371/journal.pone.0240005 (PMC7556529; doi:10.1371/journal.pone.0240005)
Supplement: Attachment — Submitted filename: Response to Editor and Reviewers.pdf [file pone.0240005.s010.pdf]

Response to Editors and Reviewers

Response to Editor

To address the editor's comments and concerns, we have made the following changes to the manuscript.

First, we have added concrete results and statistics to the abstract.

Second, regarding the editor's comment on the use of both WhatsApp and Qualtrics, no subscribers were reached through Qualtrics. All subscribers were messaged through WhatsApp and were then given the opportunity to respond to questions either through WhatsApp or Qualtrics. Qualtrics is simply a surveying platform that is commonly used by researchers. While Qualtrics allows for a smoother experience for both the surveyor and the respondent, it is hosted on a website and thus requires mobile data whereas WhatsApp does not. As such, we provided the option of taking the survey either manually through WhatsApp by typing their responses and sending it back to Kubatana, or through the external Qualtrics platform. We have briefly clarified this in the manuscript on LINES 72-77.

Third, regarding the editor's comment on the short and long experimental lists, we have added some clarifying text to the manuscript to explain the methodology more fully from LINES 120 to 133. In addition, we have added a citation to a paper which explains the approach, which is also called an 'item count technique' in other disciplines.

Fourth, we have added text and a citation to the manuscript to explain the blocking procedure from LINES 134 to 140. In short, grouping our units of treatment assignment into similar "blocks," and *then* randomizing treatment assignment within these blocks. We then use randomization block fixed effects during the estimation of treatment effects, which allows to improve the precision of such estimates.

Fifth, we have added information on statistical software, Stata 16 and R, on LINES 153 and 154.

Finally, following the indication that our ethics statement must appear in the Methods section of our manuscript, we have added a paragraph on this from LINES 92 to 98 under the Research design section.

Response to Reviewer #1

Reviewer #1 asked us to include relevant statistics on the degree to which WhatsApp is used in Zimbabwe. WhatsApp is the dominant social media platform in the country, accounting for roughly half of the internet usage overall. Competing social media platforms, such as Facebook, take up only 1% of the overall internet usage. While the exact number of users cannot be determined due to privacy concerns, an estimate based on data bundle purchasing in the country suggests at least 5.2 million WhatsApp users. We have made this point clearer on LINES 19 to 23 of the Introduction.

Response to Reviewer #2

In response to Reviewer #2's request for the number of individuals assigned to treatment and control per week, we have added these numbers on LINES 100 to 106 of the Data section of the paper. The response rate for the survey was previously included only in the tables, but we have added these to the Data section of the paper as well.

With regard to Reviewer #2's subsequent point on overlapping respondents for each survey: While we agree that assessing the overlap of respondents between the two surveys would be ideal, our (and the NGO's) prioritization of complete anonymity on these surveys meant that we had no access to individual identifiers for these surveys. We have clarified on this point on LINE 80 of the Research Design section of the paper. It is thus not possible to determine the overlap between the two weeks. However, we argue that this does not affect the validity of our results for two reasons:

1. The treatment and control groups are re-randomized each week, meaning that individuals may be in the treatment group one week and control group the next week. This means that the results we receive in the second week should be orthogonal to treatment assignment in the first week.
2. In Table 5, we show the results for Week 1 and Week 2 separately. If bias in survey responses arise in Week 2, we should observe statistically different results between Weeks 1 and 2. We find that results from Week 2 are similar to the results from Week 1, and are also similar to the results when we pool both Week 1 and Week 2, which assuages concerns about potential biases from a repeat in survey responses. We have clarified this result on LINES 195-196 under the Results section.

Finally, Reviewer #2 identifies potential demand biases arising from a survey being linked to Harvard University. We address this issue in two ways.

1. First, the surveys are completely disseminated through Kubatana, and are reaching potential respondents from a WhatsApp phone number that is recognized as being the Kubatana organization. While Kubatana notes in the survey explanation that Harvard University is helping them to analyze the effects of their messaging, the survey itself comes from Kubatana.
2. However, given that the Harvard name is still mentioned as part of the survey, and to deal with related concerns about Kubatana disseminating the survey, we deal with social desirability/demand biases through the use of the list experiment. On LINE 114 to 128, we explain how list experiments can help to guard against such biases with regard to sensitive topics such as behavior during the COVID-19 crisis. Our positive and statistically significant result on the list experiment assuages the concerns of social desirability bias in this project.
